# Effects of age on noninvasive assessments of vascular function in nonhuman primates: implications for translational drug discovery

**DOI:** 10.1186/1479-5876-11-101

**Published:** 2013-04-22

**Authors:** Delvin R Knight, Andrew H Smith, Richard L Schroeder, Chunli Huang, David A Beebe, Sharon A Sokolowski, Miao Wang

**Affiliations:** 1 Pfizer Worldwide Research and Development, Cardiovascular and Metabolic Diseases Research Unit, Pfizer Inc, Groton, CT 06340, USA

**Keywords:** Flow-mediated dilation, Pulse-wave velocity, Endothelium, Drug discovery, Thiazolidinedione, Endothelial progenitor cell, Endothelial microparticles, Nicotine

## Abstract

**Background:**

Endothelium-dependent flow mediated dilation (FMD) and pulse-wave velocity (PWV), are used as measures of vascular health and predictors of cardiovascular risk in clinical studies, and both are age-dependent. Numbers of circulating endothelial microparticles (EMPs) and endothelial progenitor cells (EPCs) are also associated with cardiovascular risk, but independent of age in humans. The use of these measurements for pre-clinical assessment of drug cardiovascular safety and efficacy in non-human primates (NHPs) may promote the translation of drug-induced effects on vascular function to clinic outcomes. However, in NHPs, the age effects on the non-invasive measurements of FMD and PWV and the relationship of EMPs/EPCs with FMD are unknown.

**Methods:**

A non-invasive, clinically-relevant approach to assess FMD and PWV was used to examine their relationship with age and with EMPs/EPCs in NHPs. The effects on FMD of nicotine and rosiglitazone were evaluated in senescent primates in an effort to validate our FMD method for pre-clinical assessment of vascular function.

**Results:**

FMD and PWV methods were established in a colony (n = 25) of metabolically healthy, cynomolgus monkeys ranging in age from 6 to 26 years. FMD, defined as the percent change, at 1 min of cuff release, from baseline vascular diameter (0.15 ± 0.03 cm), had a strong, negative correlation with age (r = -0.892, p < 0.0001), ranging from 6% to 33%. PWV positively correlated with age (r = 0.622, p < 0.002) in the same healthy monkeys. Nicotine and rosiglitazone, were evaluated in subsets of senescent primates (mean age 16.3 ± 1.5[SEM] years). Rosiglitazone significantly improved FMD (21.0 ± 1.6% vs. vehicle 16.3 ± 1.6%, p < 0.01) without changing baseline diameters, and coincided with a significant increase in circulating numbers of endothelial progenitor cells (CD45-CD31 + CD34 + VEGFR2+ 7.1 ± 1.3 vs. 4.8 ± 1.1 counts/μl) and a decrease in endothelial microparticles (CD45-CD42a-CD54+ 26.7 ± 11.1 vs. 62.2 ± 9.8 counts/μl)(p < 0.05). Conversely, FMD was significantly reduced with nicotine (8.7 ± 1.4% vs. vehicle 20.1 ± 2.2%, p < 0.05).

**Conclusions:**

Adult NHPs demonstrate the characteristic linear relationship between age and vascular function using the non-invasive clinically-related measurements of FMD and PWV. However, numbers of circulating EMPs and EPCs did not correlate with age. Endothelial function assessed with FMD, together with EMPs/EPCs assessment, may serve as a novel approach for translational research and therapeutic discovery. Age should be considered in the study design or data analyses when FMD or PWV is used in NHPs.

## Background

Clinically, vascular function may be evaluated non-invasively by measuring flow mediated vascular dilation (FMD) [1], which assesses endothelial function as arterial vasodilation induced by an increase in luminal blood flow, and pulse wave velocity (PWV), which measures arterial stiffness as the velocity of pressure pulse waves through the aorta [2]. Both endothelial dysfunction and aortic stiffness are associated with diseases and conventional cardiovascular risk factors [1-6]. FMD and PWV are also predictors of cardiovascular events in humans [7,8]. Aging is associated with both functional and structural changes in the vasculature [9], and leads to impaired FMD [6] and increased PWV [3,10]. When assessing vascular function during interventional drug trials [11,12], age is a classic confounding factor.

Non-human primate (NHP) is an important translational preclinical model for cardiovascular disease, diabetes and other metabolic diseases [13-16] and like humans can naturally develop glucose intolerance and diabetes without pharmacological or surgical inducement [16,17]. FMD and PWV have been used to evaluate test drugs and disease effects [16,18] in NHPs. However, the age effects on these measures of vascular function (FMD and PWV), which are well-documented in humans, remain unaddressed in NHPs. Since NHP can live as adults for decades, a better understanding of the relationship between age and FMD/PWV would be important in order to avoid the confounding effects of age when interpreting FMD and PWV [18].

Thus, our primary goal was to develop the methods to measure FMD and PWV in NHPs and to examine the effects of age on these clinically relevant assessments of vascular function. In addition, we explored using FMD in senescent NHPs as a translational approach to cardiovascular efficacy/safety assessment. Here, we focus on FMD because there is limited information on using PWV for clinical assessment of drug effects, and FMD has emerged as an important clinical measurement for assessing cardiovascular risk factors and disease therapies [5,11,19]. For example, chronic treatment with rosiglitazone improve FMD from ~5% to ~7% in patients with type 2 diabetes mellitus [11]. To make the efficacy/safety assessment, rosiglitazone, which targets PPARγ nuclear receptor, was used to show beneficial vascular effects, and nicotine was used to mimic the well-known detrimental effects of smoking on FMD. Finally, endothelial microparticles (EMPs, membrane coated vesicles) and endothelial progenitor cells (EPCs, a population of cells in the blood involved in endothelial repair), are associated with cardiovascular risk in humans [20,21] and are being investigated as clinical biomarkers of endothelial vascular health. Here, we have a unique opportunity to examine the relationship of EPCs/EMPs with age and FMD by addressing these “clinically relevant” biomarkers in our primate model. Since PPARγ improves endothelial functions [22,23] and increases the numbers of EPCs [24,25], we determined the effects of rosiglitazone on circulating EMPs and EPCs, as well as, FMD in NHPs.

## Methods

### Animals

Studies were conducted with the approval of the Pfizer Institutional Animal Care and Use Committee. Twenty-five healthy monkeys (*Macaca fascicularis*, also known as cynomolgus) (aged at 6 to 26 years, 18 males and 7 females) were studied. These monkeys were paired housed, when appropriate, in a room with temperature of 19-25°C, humidity of 30-70%, and 12:45/11:15 light/dark cycles with a 45 minute ramping period to full lights on/off as simulated dawn/dusk. The monkeys were fed a primate chow diet (PMI Certified Hi Fiber Primate Diet, LabDiet 5K91, PMI Nutrition International, St. Louis, MO) supplemented with fruits and/or vegetables with the amount adjusted to maintain a normal body weight. Conscious primates, previously conditioned to chair restraint (Plas-Labs, Inc. Lansing, MI), were seated comfortably in a primate chair for drug administration and blood collection prior to vascular measurements.

### Measurement of artery flow mediated dilation (FMD) and pulse-wave velocity (PWV)

**Figure 1:**
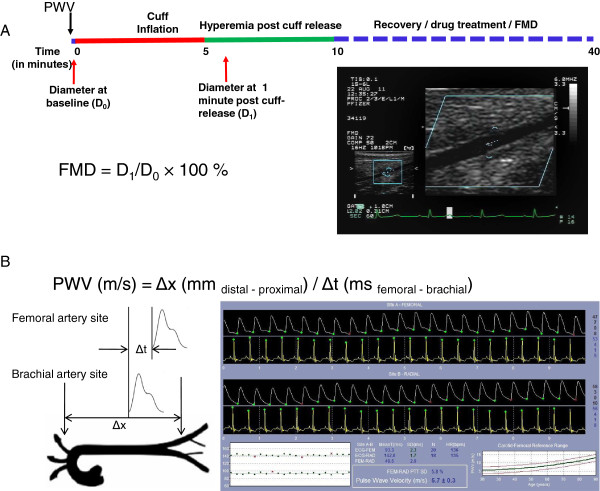
**Noninvasive assessments of vascular function in nonhuman primates(NHPs). A**: FMD measurement in NHPs. A study protocol diagram is shown in panel **A**. Primates were sedated, and PWV was measured first, followed by baseline femoral diameter measurement (D_0_). Following a 5-min ischemia, the reactive hyperemia response was triggered by cuff release, and vessel diameter was measured at 1 min of the hyperemia response (D_1_). Following a recovery from 1^st^ FMD determination, additional drug treatment and 2^nd^ FMD measurement may apply if needed, and this is typically achieved within 40 min. FMD is calculated as percentage change of D_1_ from D_0_. An ultrasonographic picture is shown that depicts a femoral artery. **B**: PWV measurement in NHPs. Pulse waves from the right brachial and femoral arteries, as depicted, were collected. PWV was calculated as the Distance/Time using the equation shown, which is based on distance difference (Δx) between the two detection sites (femoral artery and brachial artery) and Δt, the difference in time lapse of a pressure wave gated to the corresponding ECG R-wave at the two detection sites (B left panel). A window of PWV recording by SphygmoCor program is shown (**B** right panel). See Method part for detailed description.

We established clinically-relevant, non-invasive methods to examine FMD and PWV in non-human primates (Figure 1). FMD and PWV were measured between 9 am to 12:30 pm on overnight fasted monkeys. Monkeys were sedated with butorphanol (0.3 mg/kg, im) and midazolam (0.5 mg/kg, im), or with ketamine (5 mg/kg, im) + midazolam (0.35 mg/kg, im), placed on a 37°C heating pad to maintain body temperature, oriented in a right lateral recumbent position to allow access the right brachial and femoral arteries. Stable mean arterial blood pressure and heart rate during these procedures averaged 79 mmHg and 142 beats/min, respectively.

Electrocardiography (ECG) electrodes were placed, to collect a lead II ECG signal. Pulse waves from the brachial and femoral arteries, were recorded using a SphygmoCor (AtCor, Itasca, IL) PWV probe that was associated with a modified SphygmoCor program (CvMSV9; modified for high rates). Distal-proximal distances were measured and entered into the program. PWV was calculated by the program using the equation PWV = Δ*x*/Δ*t*, where Δ*x* is difference in distance between the two sites relative to the sternal notch, and Δ*t* is the time for the wave to travel that distance (the difference in time lapse from R-wave peak to pressure wave peak, between the two sites). A minimum of 2 measurements were used to calculate average PWV. Brachial artery measurements were taken at the axilla and femoral artery measurements were taken mid-thigh for PWV. The average distal distance (femoral to notch) was 338 ± 5.4 mm and average proximal distance (brachial to notch) was 75 ± 3.1 mm (n = 25). The distal vs. proximal distance allowed sufficient difference to calculate the PWV. Average detection variation for PWV was 10.6%.

FMD in the NHPs was measured using ultrasonography, as illustrated in the Figure 1A. The right leg was positioned and gently restrained to prevent movement during the study. An infant V-Lok cuff (WA Baum, Copiague, NY) was then placed below the knee and connected to a manometer. After applying ultrasonic gel, a linear array probe (6–15 MHz), connected to a Hewlett Packard Sonos® 5500 and mounted on an articulated arm (PPM100, Tektronix, Inc. Beaverton, OR), was positioned over the femoral artery, manipulated until a clear signal was obtained and then locked in place for the remainder of the protocol. Once a baseline recording of the femoral artery was collected, the cuff was inflated within 2 s to a super-systolic pressure (290-300 mmHg). After 5 min the cuff pressure was rapidly released (<1 sec.) and a second recording was collected at 1 minute of reperfusion approximating the time of maximal flow mediated dilation [16]. Vessel diameter was measured, using electronic calipers, for baseline and 1 minute recordings. The average baseline femoral artery diameter for the study colony was 0.151 ± 0.03 cm (n = 25). Flow mediated dilation was calculated as the% change from baseline diameter. Each diameter measurement was the average of 4 segmental measurements taken ~1 mm apart gated to the ECG T wave. At the end of the experiment, monkeys were recovered by naloxone (0.05 mg/kg im and 0.05 mg/kg iv – if they received butorphanol) and flumazenil (2 mL per monkey, iv). FMD conducted 15 min apart showed average variation <5.5%.

### Study design

Six fasted monkeys (17 ± 1 years of age) were treated orally with both vehicle and rosiglitazone (1 mg/kg, coated on one PrimaTreat; Bio-Serv, Frenchtown, NJ) ≥2 weeks apart in a 2 × 2 crossover design (2 in vehicle-drug sequence and 4 in drug-vehicle sequence). Drug or vehicle was administered at 6:00 AM, and, FMD and PWV were measured ~ six hours later, a time point that showed increased FMD in a human study [12]. Blood samples were collected (via cephalic vein) just prior to sedation, and immediately processed for determination of endothelial microparticle, endothelial progenitor cell and TNFα (detailed in following methods). These treatments were repeated in the same animals to determine the effect of rosiglitazone on lipids and intravenous glucose tolerance test.

A separate group of monkeys (n = 3, 15 ± 4 years of age) were sedated, and intravenously infused with saline vehicle, then with 0.844 μg/kg/min nicotine bitartrate (Sigma Aldrich, St. Louis, MO), at rate of 0.4 mL/min. FMD was recorded at 5 min of vehicle infusion, and at 15 min of nicotine infusion (while the nicotine infusion was constantly maintained), and the study was completed within 35 min. The nicotine dose used was calculated to approximate the predicted blood nicotine concentration (40 nM) of a human study in which chewing nicotine gum reduced FMD [26,27].

### Endothelial microparticle (EMP) and endothelial progenitor cell (EPC) sample preparation and analysis

To measure EPC, whole blood was collected into EDTA tubes from animals fasted overnight and, incubated with anti-human CD34, CD31, CD45 (Becton Dickinson and Co./BD, Franklin Lakes, NJ) and VEGFR2 (R&D Systems; Minneapolis, MN.) antibodies. After lysing the red blood cells, the samples were analyzed using a high performance Canto II flow cytometer (BD) with DIVA software (BD).

At the same time, whole blood was collected in sodium citrate tubes and platelet poor plasma (PPP) was prepared to measure EMPs. An aliquot of PPP was added to each of the two panels of antibodies: Panel 1 [CD42a-APC (conjugated in house), CD31-FITC, CD45-AF700, Annexin V-V450, CD105-PerCP (BD), CD54-PE (Invitrogen)]; Panel 2 [CD42a-APC (conjugated in house), CD31-FITC, CD45-AF700, CD106-PE-Cy7 (conjugated in house), CD62E-PE (BD), VEGFR2-PerCP (R&D Systems), CD144-Pacific Blue (conjugated in house) (BioLegend, San Diego, CA.)]. Cytofix was then added and a 500 uL aliquot was transferred into a Trucount tube (BD) and analyzed by flow cytometry.

### Biochemical tests and cytokine determination

Venous blood samples were collected in serum separator tubes from untreated, rosiglitazone treated or vehicle treated monkeys fasted overnight, centrifuged at 3000 rpm for 10 min at 4°C, and levels of lipids and glucose measured on Siemens Advia 2400 chemistry analyzer, and levels of insulin measured on the Siemens Centaur Immunoassay, with kits of Advia Chemistry Systems (Siemens Healthcare Diagnostics Inc., Tarrytown, NY).

Lipopolysaccharide (LPS, Sigma-Aldrich) stimulated release of TNFα was carried out, ex vivo, using sodium heparin anti-clotted whole blood that was harvested 6 hours after rosiglitazone dosing. Blood was stimulated with 10 ng/mL LPS for six hours with gentle rocking, plasma separated by 14,000 rpm centrifugation for 20 min at 10-15°C, and TNFα measured by using Linco Luminex®mouse cytokine assay(EMD Millipore, Billerica, MA).

### Intravenous glucose tolerance test (IVGTT)

Monkeys fasted overnight were sedated with ketamine for duration of IVGTT. Two iv catheters were placed for blood sampling and glucose administration, respectively. Baseline samples were drawn starting approximately 10 min after initial ketamine dose and catheter placement. A 50% dextrose solution was then administered, iv, at 250 mg/kg over 20 s. Blood samples (~1 mL) for glucose and insulin analysis were collected in serum tubes at 2, 5, 7, 10, 15, 20 and 30 min post dextrose administration, centrifuged and the resulting serum analyzed for glucose/insulin levels. The Area Under the Curve (AUC) for plasma glucose and insulin levels from 0 to 30 min was calculated. Glucose disappearance rate was expressed as K_glc_, the reduction of the log_e_ of plasma glucose levels between 5 & 20 min following iv glucose administration and calculated using the formula: K_glc_ = (log_e_G_1_ – log_e_G_2_)/(t_2_ – t_1_) where G_1_ and G_2_ are the glucose levels at time 5 min (t_1_) and 20 min (t_2_) [28].

### Statistical analysis

Pearson correlation coefficient was computed for correlation study. Linear regression models were applied to investigate the relationships of age with FMD and PWV and the relationship between FMD and PWV. In order to evaluate the treatment effect of rosiglitazone on the endpoints of interest (FMD and PWV) compared to vehicle, a linear mixed effect model was applied to analyze each of the two endpoints from this 2 × 2 crossover study with age adjustment. Specifically, the model has treatment (drug vs. vehicle), sequence (1 vs. 2), and period (1 vs. 2) as fixed effects, age as a covariate, and subject (monkey) as random effect. Paired *t* test was used to compare EMPs/EPCs pre- and post- rosiglitazone treatment. Paired *t* test was applied to analyze the FMD data measured in the nicotine infusion study to evaluate the effect of nicotine on FMD compared to saline vehicle. Results are presented as parameter estimate (Mean) ± SEM. A two-sided p < 0.05 was considered statistically significant.

## Results

### Age correlates to FMD and PWV in NHPs

To examine age effects on FMD and PWV in NHPs, we established clinically-relevant, non-invasive methods (Figure 1). FMD was measured in a colony of metabolically healthy monkeys (n = 25) with age ranging from 6-26 years. Serum levels of lipids (total cholesterol, HDL, LDL, and triglycerides; Additional file 1: Table S1A) showed no indication of hyperlipidemia, and levels of blood urea nitrogen and creatinine were normal indicating normal renal function. Since cynomolgus monkeys can naturally become glucose intolerant the primates were assessed using an IVGTT. Fasted blood glucose levels were normal and the areas under the curve for glucose and the disappearance of glucose in response to dextrose challenge were also normal, indicating no glucose intolerance in these monkeys (Additional file 1: Table S1B).

**Figure 2:**
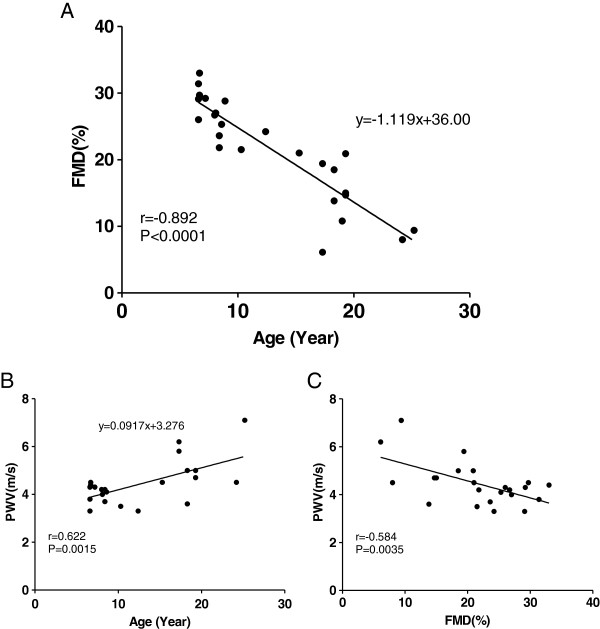
**Age correlates to FMD and PWV in NHPs.** Age strongly correlates with FMD (**A**: r = -0.892, p < 0.0001 n = 25) and with PWV (**B**: r = 0.622, p < 0.002, n = 23). FMD negatively correlates with PWV (**C**: r = -0.584, p < 0.002, n = 23). Two monkeys were excluded from the PWV analysis: one with a poor positional signal and one that was (low) out of range.

In these metabolically healthy monkeys, FMD demonstrated a robust dynamic range (6 to 33%) that correlated negatively with age (r = -0.892, p < 0.0001; regression equation: y(%FMD) = -1.119x(year) + 36.00) (Figure 2A). These data indicate that age has a significant impact on FMD in NHPs. In the same monkeys, PWV ranged from 3 to 7 m/s, and also correlated with age (r = 0.622, p < 0.002; regression equation: y(PWV) = 0.0917x(year) + 3.276) (Figure 2B). The reduction in FMD also correlated with increase in PWV(r = -0.584, p < 0.002) (Figure 2C). However, unlike FMD and PWV, age did not significantly correlate with numbers of EPCs or subsets of EMPs in these monkeys (Additional file 2: Table S2).

### Rosiglitazone improved, while nicotine impaired, FMD in metabolically healthy monkeys

**Figure 3:**
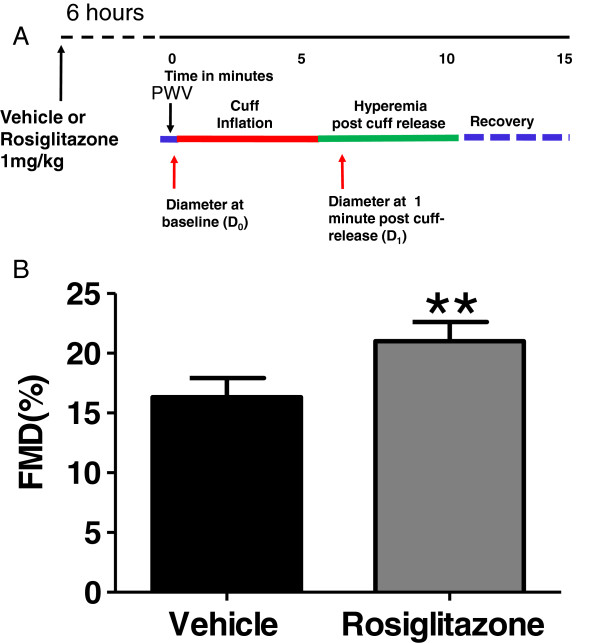
**Acute treatment of rosiglitazone (panel A, study protocol, see Methods for details) improves FMD in NHPs (panel B).** Results were adjusted by age. n = 6; **: p < 0.01.

Using senescent monkeys, we examined the effect of rosiglitazone on FMD. The FMD was significantly increased following a single oral dose of rosiglitazone (21.0 ± 1.6% vs. vehicle 16.3 ± 1.6%) (Figure 3). Rosiglitazone treatment had no effect on baseline femoral artery diameter (1.52 ± 0.04 vs.1.51 ± 0.04 mm, respectively), and did not affect PWV (Additional file 3: Figure S1) when compared to vehicle treatment. In addition, when compared to vehicle treatment, this dose of rosiglitazone had no significant metabolic effects on baseline glucose, insulin, lipids, IVGTT responses, or anti-inflammatory effects, as indicated by the lack of LPS-stimulated TNFα release in these metabolically healthy primates (Additional file 4: Table S3). However, rosiglitazone treatment significantly increased EPCs (CD45-CD31 + CD34 + VEGFR2+ 7.1 ± 1.3 vs. 4.8 ± 1.1 counts/μl) and reduced EMPs (CD45- CD42a- CD54+ 26.7 ± 11.1 vs. 62.2 ± 9.8 counts/μl) (Additional file 3: Figure S2). CD45- CD42a- CD105 + EMPs (16.5 ± 3.7 vs. 33.2 ± 5.6 counts/μl, n = 5, p = 0.06) and CD45- CD42a- CD144+ EMPs (5.0 ± 1.5 vs. 41.8 ± 17.6 counts/μl, n = 5, p = 0.09) also trended lower.

**Figure 4:**
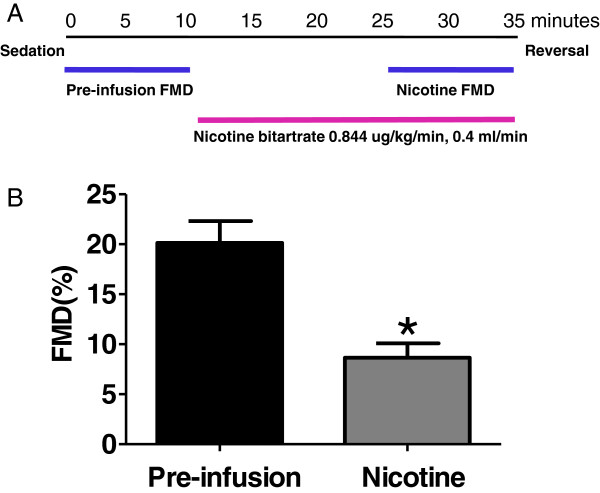
**Nicotine infusion (panel A, study protocol, see Methods for details) impairs FMD in NHPs (panel B).** n = 3; *: p < 0.05.

We next examined whether the deleterious effect of nicotine on FMD can be detected in NHPs. Intravenous infusion of nicotine in healthy monkeys significantly reduced FMD (8.7 ± 1.4% vs. vehicle 20.1 ± 2.2%) (Figure 4). The plasma level of nicotine, at the time of FMD measurement, was ~27 nM.

## Discussion

### Non-invasive measurement of vascular function in NHPs

We established a non-invasive method for FMD to examine the effects of age on vascular function in NHPs. Our protocol to measure FMD in NHP was adapted from human protocols [1,7,29,30] and as such, FMD was measured following a hyperemic response triggered by distal limb ischemia. An important aspect of the present technique was the use of a combination of anesthetics (midazolam and butorphanol or ketamine) to keep the muscle relaxed without limb movement that was not observed with ketamine alone. Measuring FMD using the articulating arm also obviates the need to manipulate the probe while measuring vascular diameter. These refinements were key in allowing us to detect a dynamic range of changes in FMD associated with age and pharmacological interventions in NHPs.

### Age and FMD

Previously, total peripheral resistance was shown to fall less with acetylcholine infusion in 19.8 ± 0.6 years old monkeys than in 7.1 ± 0.4 years old monkeys [31], suggesting an age-related impairment of vascular dilatory response to acetylcholine. Since NHP can live as adults for decades, a better understanding of the relationship between age and FMD/PWV would be important in order to avoid the confounding effects of age. Here, we examined the age effect on the flow-induced vascular dilatory response in a colony of adult, sexually mature monkeys, 6 to 26-years of age. The robust age-FMD relationship (Figure 2A) has at least three implications. (i) The age-dependent nature of FMD recapitulates that seen in human [6], suggesting a translational feature of NHP model of FMD. (ii) For studies evaluating the effects of test agents on FMD in NHPs, prior randomization by age and/or recording of age at baseline is necessary, and age-dependent vs. disease-ameliorating effects on FMD may also need to be considered to clarify mechanisms of action. (iii) Prior vascular health assessment by FMD can be used to select NHPs most likely affected by test agents. This is particularly important for selecting study subjects from a heterogeneous population of NHPs. For example, monkeys develop diabetes and metabolic syndrome at varied age [16,17], and diabetes itself features endothelial dysfunction (reduced FMD) in human [32]. In addition, the age-FMD relationship allows us to put in perspective the effect of test agents on vascular function (FMD). It is interesting to note that the average improvement in FMD following rosiglitazone treatment (Figure 3) was equivalent to improving the monkeys’ vascular age by 5.2 years.

PWV also correlates with age in NHPs (Figure 2), indicating that, similar to the FMD assessment, age effect should also be considered when evaluating PWV, by prior randomization of study groups or by age adjustment. Furthermore, age-adjustment decreases variation in FMD and PWV by 79.57% and 38.67%, respectively, among these NHPs. This suggests a stronger age impact on FMD than PWV.

### Evaluation of FMD changes in NHPs

We established that the non-invasive methodology can be used to assess beneficial (rosiglitazone) and detrimental (nicotine) FMD changes following drug intervention in NHPs (Figures 3 and 4). Chronic treatment of diabetic patients with rosiglitazone ameliorates insulin resistance and its related metabolic disorder while improving FMD [11]. Acute treatment also results in FMD improvement in healthy subjects in the absence of associated metabolic effects [12]. In line with these earlier studies, we reported here that rosiglitazone improved FMD in metabolically healthy NHPs (Figure 3), without drug-related metabolic or anti-inflammatory effects (Additional file 4: Table S3). In a recent study [16] using NHPs of similar age to those studied here, 6-week pioglitazone treatment improves metabolic parameters, and reverses the impaired FMD that co-emerged with the metabolic syndrome. In senescent euglycemic monkeys, our results show that rosiglitazone caused an acute improvement in FMD that was independent of effects on cytokine (TNFα) release or metabolic effects on glucose and lipids. This suggests that PPARγ agonists may have a direct action on the endothelium in vivo beyond its systemic metabolic effects.

Despite the lack of age-related correlation of EPCs and any of the EMPs, acute treatment with rosiglitazone decreased the number of EMP(CD45-CD42a-CD54+) and increased EPC(CD45-CD31 + CD34 + VEGFR2+) (Additional file 3: Figure S2). CD54(ICAM-1) is an intercellular adhesion molecule expressed on endothelial cells, and is associated with increased cell activation [33]. Although somewhat controversial, EPCs are believed to play an important role in the endogenous repair mechanisms for maintaining the integrity of the endothelial monolayer [34,35]. PPARγ is known to regulate endothelial survival and function [22,23], as well as, EPC function [24,25]. The rosiglitazone induced increase in FMD coincided with acute increases in EPCs and suppression of EMPs suggesting an improvement in endothelial health. While these changes in EPCs and EMPs do not provide a causal, mechanistic link for the FMD changes, from a drug discovery view point, our results could provide the impetus for further studies to evaluate the use of EPCs/EMPs as biomarkers to potentially capture a signal of vasculature change of test drugs.

Cigarette smoking is associated with impairment of FMD [1,36,37], and treatment with nicotine gum attenuates FMD in human subjects [26]. In this study, the plasma levels of infused nicotine(~27 nM), at the time of FMD measurement, matched to the levels in humans that are associated with reduced FMD [37], and also approximated the nicotine level (~40 nM) predicted from another human study, in which nicotine gum treatment reduces FMD [26]. Nicotine’s effect on the cardiovascular system is complex [38], including release of pro-inflammatory mediators, thrombosis, decreasing oxygen supply, and increasing sympathetic nerve activity [39]. The significant reductions in FMD with nicotine further support the translation of this model to clinical outcomes and illustrates how robust this preclinical, NHP model is in detecting changes in FMD (only 3 animals required) in a highly controlled laboratory setting.

Unlike other models [40], the NHP is considered as a highly translational animal model of cardiovascular disease [13], and is often used as a preclinical model to evaluate therapeutic agents. This translation is also supported by the observed correlation between age and FMD/PWV in this study. NHPs are phylogenetically closer to humans, and may be particularly useful when a drug target does not exist in other species (for example, Cholesteryl ester transfer protein, a regulator of HDL levels, is not present in rodents). The non-invasive nature of FMD allows reuse of animals for cross-over and longitudinal studies. Further, the pharmacological effects on FMD might be detected with a relatively small group of animals (for example, 6 monkeys were used in the rosiglitazone study) under a well-controlled environment that is difficult to duplicate in clinical studies. The described FMD methodology using NHPs, might be highly valuable both for translational research, drug efficacy/safety evaluation, and for clinical dose-selection. This is particularly important for developing diabetes drugs. New guidelines of FDA require cardiovascular safety assessment before approval of any diabetes drugs [41].

### Study limitations

Blood flow was not assessed during these FMD determinations. FMD measured under our current protocol does not distinguish between macro- and micro-circulation effects; rather, it serves as an integrated assessment of vascular endothelium function. The variability of PWV detection under current protocol needs to be optimized, which may limit the power to detect a biological impact. Detection of EMPs and EPCs may have been limited by the availability of antibodies and the degree of cross-species reactivity of the human antibodies with monkey. As they become available, the use of additional antibodies for EMP/EPC markers and their functional characterization could provide further insight and utility in assessing vascular health.

## Conclusions

In conclusion, we established a non-invasive, clinically-relevant approach to assess vascular function in NHP by measuring FMD and PWV, and identified age as a significant modifier of FMD, and to a less degree, of PWV. Acute treatment with rosiglitazone improves FMD, while nicotine reduces FMD in senescent primates. Senescent primates with age-related endothelial dysfunction may provide a unique model for evaluating preclinically the efficacy and safety of therapeutic agents on endothelial function (i.e. FMD). The endothelial function assessment with FMD in NHPs and the associated circulating biomarkers (i.e. EPC, EMP), as described herein, may serve as a novel approach for translational research and therapeutic discovery. Like in humans, age is an important confounder that should be considered when assessing cardiovascular health and interpreting drug actions in NHPs.

## Abbreviations

FMD: Flow mediated dilation; PWV: Pulse-wave velocity; NHP: Non-human primate; EMP: Endothelial microparticle; EPC: Endothelial progenitor cell; PPARγ: Peroxisome proliferator-activated receptor gamma; IVGTT: Intravenous glucose tolerance test.

## Competing interests

The authors declare that they have no competing interests. DRK’s current address is National Institutes of Health, Department of Health and Human Services. The opinions expressed in this article are the authors’ own and do not reflect the view of the National Institutes of Health, the Department of Health and Human Services, or the United States government.

## Authors’ contributions

DRK designed and supervised the study while employed at Pfizer, Inc., and RLS and AHS performed the in vivo study. MW designed the nicotine study and the cytokine study. DAB and MW assisted with the in vivo study setup and sample processing. SAS and CH designed the EMP and EPC analysis, CH measured the EMPs and EPCs, and SAS analyzed and interpreted the EMP and EPC data. DRK, AHS, and MW analyzed and interpreted the in vivo and in vitro data. MW wrote the manuscript. All authors have read and approved the manuscript for publication.

## Supplementary Material

Additional file 1: Table S1 Basal metabolic parameters of the study monkeys. A: Blood lipids profile. B: Glucose, insulin, and IVGTT. Click here for file

Additional file 2: Table S2 FMD or age does not correlate with numbers of EPCs or EMPs in metabolically healthy monkeys. n = 19 and 21 for EPC and EMP, respectively. Click here for file

Additional file 3: Figure S1 Rosiglitazone does not affect PWV in NHPs. n = 6, p = 0.22 (age adjusted). NS: non-significant. **Figure S2.** Rosiglitazone results in favorable changes in circulating numbers of EMPs (CD45-CD42a-CD54+) (A) and EPCs (CD45-CD31 + CD34 + VEGFR2+) (B) in euglycemic, senescent monkeys. n = 5, *: p < 0.05; **: p < 0.01. Click here for file

Additional file 4: Table S3 Effect of rosiglitazone treatment on blood biochemistry and LPS-stimulated TNFα release. No significant difference between the two groups, n = 5 (vehicle) or 6 (rosiglitazone). K_glc, 5-20_: slope of the disappearance of glucose 5-20 min after dosing; AUC_0-30_: area under the curve for glucose or insulin (0-30 min). Click here for file

## References

[B1] Celermajer DS Non-invasive detection of endothelial dysfunction in children and adults at risk of atherosclerosis Lancet 1992 340 8828 1111 1115 10.1016/0140-6736(92)93147-F 1359209

[B2] Wilkinson IB Cockcroft JR Webb DJ Pulse wave analysis and arterial stiffness J Cardiovasc Pharmacol 1998 32 Suppl 3 S33 S37 9883745

[B3] Avolio AP Effects of aging on changing arterial compliance and left ventricular load in a northern Chinese urban community Circulation 1983 68 1 50 58 10.1161/01.CIR.68.1.50 6851054

[B4] Halcox JP Endothelial function predicts progression of carotid intima-media thickness Circulation 2009 119 7 1005 1012 10.1161/CIRCULATIONAHA.108.765701 19204308

[B5] Charakida M Assessment of atherosclerosis: the role of flow-mediated dilatation Eur Heart J 2010 31 23 2854 2861 10.1093/eurheartj/ehq340 20864485

[B6] Celermajer DS Endothelium-dependent dilation in the systemic arteries of asymptomatic subjects relates to coronary risk factors and their interaction J Am Coll Cardiol 1994 24 6 1468 1474 10.1016/0735-1097(94)90141-4 7930277

[B7] Yeboah J Brachial flow-mediated dilation predicts incident cardiovascular events in older adults: the Cardiovascular Health Study Circulation 2007 115 18 2390 2397 10.1161/CIRCULATIONAHA.106.678276 17452608

[B8] Vlachopoulos C Aznaouridis K Stefanadis C Prediction of cardiovascular events and all-cause mortality with arterial stiffness: a systematic review and meta-analysis J Am Coll Cardiol 2010 55 13 1318 1327 10.1016/j.jacc.2009.10.061 20338492

[B9] Lakatta EG Cardiovascular regulatory mechanisms in advanced age Physiol Rev 1993 73 2 413 467 8475195 10.1152/physrev.1993.73.2.413

[B10] Mattace-Raso FU Determinants of pulse wave velocity in healthy people and in the presence of cardiovascular risk factors: ‘establishing normal and reference values’ Eur Heart J 2010 31 19 2338 2350 20530030 10.1093/eurheartj/ehq165 PMC2948201

[B11] Kelly AS Rosiglitazone improves endothelial function and inflammation but not asymmetric dimethylarginine or oxidative stress in patients with type 2 diabetes mellitus Vasc Med 2007 12 4 311 318 10.1177/1358863X07084200 18048467

[B12] Walcher T Rapid effect of single-dose rosiglitazone treatment on endothelial function in healthy men with normal glucose tolerance: data from a randomised, placebo-controlled, double-blind study Diab Vasc Dis Res 2010 7 3 178 185 10.1177/1479164110367812 20460360

[B13] Shelton KA Kaplan TBCJR Nonhuman Primate Models of Atherosclerosis Nonhuman Primates in Biomedical Research, Volume Chapter 8 2012 Second 385 411

[B14] McLenachan JM Loss of flow-mediated endothelium-dependent dilation occurs early in the development of atherosclerosis Circulation 1991 84 3 1273 1278 10.1161/01.CIR.84.3.1273 1884452

[B15] Howard TD Epigenetic changes with dietary soy in cynomolgus monkeys PLoS One 2011 6 10 e26791 10.1371/journal.pone.0026791 22046358 PMC3201974

[B16] Zhang X Rhesus macaques develop metabolic syndrome with reversible vascular dysfunction responsive to pioglitazone Circulation 2011 124 1 77 86 10.1161/CIRCULATIONAHA.110.990333 21690491 PMC3775509

[B17] Wagner JD Naturally occurring and experimental diabetes in cynomolgus monkeys: a comparison of carbohydrate and lipid metabolism and islet pathology Toxicol Pathol 2001 29 1 142 148 10.1080/019262301301418955 11215678

[B18] Farrar DJ Anatomic correlates of aortic pulse wave velocity and carotid artery elasticity during atherosclerosis progression and regression in monkeys Circulation 1991 83 5 1754 1763 10.1161/01.CIR.83.5.1754 2022028

[B19] Moens AL Flow-mediated vasodilation: a diagnostic instrument, or an experimental tool? Chest 2005 127 6 2254 2263 10.1378/chest.127.6.2254 15947345

[B20] Werner N Circulating endothelial progenitor cells and cardiovascular outcomes N Engl J Med 2005 353 10 999 1007 10.1056/NEJMoa043814 16148285

[B21] Bernal-Mizrachi L High levels of circulating endothelial microparticles in patients with acute coronary syndromes Am Heart J 2003 145 6 962 970 10.1016/S0002-8703(03)00103-0 12796750

[B22] Kanda T PPARgamma in the endothelium regulates metabolic responses to high-fat diet in mice J Clin Invest 2009 119 1 110 124 19065047 10.1172/JCI36233 PMC2613459

[B23] Werner C Pioglitazone activates aortic telomerase and prevents stress-induced endothelial apoptosis Atherosclerosis 2011 216 1 23 34 10.1016/j.atherosclerosis.2011.02.011 21396644

[B24] Liang C Rosiglitazone via upregulation of Akt/eNOS pathways attenuates dysfunction of endothelial progenitor cells, induced by advanced glycation end products Br J Pharmacol 2009 158 8 1865 1873 10.1111/j.1476-5381.2009.00450.x 19917066 PMC2807648

[B25] Sorrentino SA Oxidant stress impairs in vivo reendothelialization capacity of endothelial progenitor cells from patients with type 2 diabetes mellitus: restoration by the peroxisome proliferator-activated receptor-gamma agonist rosiglitazone Circulation 2007 116 2 163 173 10.1161/CIRCULATIONAHA.106.684381 17592079

[B26] Rudolph TK Myeloperoxidase deficiency preserves vasomotor function in humans Eur Heart J 2011 33 13 1625 1634 21724624 10.1093/eurheartj/ehr193 PMC3388013

[B27] Hukkanen J Jacob P 3rd Benowitz NL Metabolism and disposition kinetics of nicotine Pharmacol Rev 2005 57 1 79 115 10.1124/pr.57.1.3 15734728

[B28] Jen K-LC Hansen BC Glucose disappearance rate in rhesus monkeys: Some technical considerations Am J Primatol 1988 14 2 153 166 10.1002/ajp.1350140206 31973454

[B29] Rossi R Prognostic role of flow-mediated dilation and cardiac risk factors in post-menopausal women J Am Coll Cardiol 2008 51 10 997 1002 10.1016/j.jacc.2007.11.044 18325438

[B30] Gaenzer H Flow-mediated vasodilation of the femoral and brachial artery induced by exercise in healthy nonsmoking and smoking men J Am Coll Cardiol 2001 38 5 1313 1319 10.1016/S0735-1097(01)01575-3 11691501

[B31] Asai K Peripheral vascular endothelial dysfunction and apoptosis in old monkeys Arterioscler Thromb Vasc Biol 2000 20 6 1493 1499 10.1161/01.ATV.20.6.1493 10845863

[B32] Feng B Circulating level of microparticles and their correlation with arterial elasticity and endothelium-dependent dilation in patients with type 2 diabetes mellitus Atherosclerosis 2010 208 1 264 269 10.1016/j.atherosclerosis.2009.06.037 19674745

[B33] Jimenez JJ Endothelial cells release phenotypically and quantitatively distinct microparticles in activation and apoptosis Thromb Res 2003 109 4 175 180 10.1016/S0049-3848(03)00064-1 12757771

[B34] Urbich C Dimmeler S Endothelial progenitor cells: characterization and role in vascular biology Circ Res 2004 95 4 343 353 10.1161/01.RES.0000137877.89448.78 15321944

[B35] Foteinos G Rapid endothelial turnover in atherosclerosis-prone areas coincides with stem cell repair in apolipoprotein E-deficient mice Circulation 2008 117 14 1856 1863 10.1161/CIRCULATIONAHA.107.746008 18378610

[B36] Celermajer DS Cigarette smoking is associated with dose-related and potentially reversible impairment of endothelium-dependent dilation in healthy young adults Circulation 1993 88 5 Pt 1 2149 2155 8222109 10.1161/01.cir.88.5.2149

[B37] Kallio K Tobacco smoke exposure is associated with attenuated endothelial function in 11-year-old healthy children Circulation 2007 115 25 3205 3212 10.1161/CIRCULATIONAHA.106.674804 17548727

[B38] Glantz SA Parmley WW Passive smoking and heart disease. Mechanisms and risk JAMA 1995 273 13 1047 1053 10.1001/jama.1995.03520370089043 7897790

[B39] Najem B Acute cardiovascular and sympathetic effects of nicotine replacement therapy Hypertension 2006 47 6 1162 1167 10.1161/01.HYP.0000219284.47970.34 16651463

[B40] Libby P Ridker PM Hansson GK Progress and challenges in translating the biology of atherosclerosis Nature 2011 473 7347 317 325 10.1038/nature10146 21593864

[B41] Center for Drug Evaluation and Research, F.a.D.A Guidance for Industry, Diabetes Mellitus — Evaluating Cardiovascular Risk in New Antidiabetic Therapies to Treat Type 2 Diabetes www.fda.gov/downloads/Drugs/GuidanceComplianceRegulatoryInformation/Guidances/UCM071627.pdf, 200822797986

