# Supplementary material for: Effects of age on noninvasive assessments of vascular function in nonhuman primates: implications for translational drug discovery
Source: J Transl Med. 2013 Apr 22;11:101. doi: 10.1186/1479-5876-11-101 (PMC3644259; doi:10.1186/1479-5876-11-101)
Supplement: Additional file 1: Table S1 — Basal metabolic parameters of the study monkeys. A: Blood lipids profile. B: Glucose, insulin, and IVGTT. [file 1479-5876-11-101-S1.doc]

**Table S1**: Basal metabolic parameters of the study monkeys.

**A: Blood lipids profile**

| Fasted | Mean ± SEM | Range | Ref. Range |
| --- | --- | --- | --- |
| Total cholesterol (mg/dL) | 91.2 ± 2.5 | 59 - 111 | 69 - 139 |
| HDL- cholesterol (mg/dL) | 49.4 ± 2.3 | 29 - 70 | 54 - 93 |
| LDL- cholesterol (mg/dL) | 33.1 ± 1.5 | 19 - 52 | 32 - 64 |
| Triglycerides (mg/dL) | 58.8 ± 6.4 | 21 - 131 | 32 - 132 |

**B : Glucose, insulin, and IVGTT**

| Fasted | | Mean ± SEM | Normal values (in-house) |
| --- | --- | --- | --- |
| Glucose (mg/dL) | | 63 ± 2 | <80 |
| Insulin (uU/mL) | | 7 ± 1 | <18 |
| IVGTT Responses | Glucose (AUC_0-30_) | 2082 ± 100 | <4000 |
|  | IVGTT K_glc, 5-20_ | 3.74 + 0.19 | >2 .0 |

K_glc, 5-20_: slope of the disappearance of glucose 5-20 minutes after dosing; AUC_0-30_: area under the curve for glucose (0-30 minutes).
