# Supplementary material for: Effects of age on noninvasive assessments of vascular function in nonhuman primates: implications for translational drug discovery
Source: J Transl Med. 2013 Apr 22;11:101. doi: 10.1186/1479-5876-11-101 (PMC3644259; doi:10.1186/1479-5876-11-101)
Supplement: Additional file 2: Table S2 — FMD or age does not correlate with numbers of EPCs or EMPs in metabolically healthy monkeys. n = 19 and 21 for EPC and EMP, respectively. [file 1479-5876-11-101-S2.doc]

**Additional file 2 Table S2:** FMD or age does not correlate with numbers of EPCs or EMPs in metabolically healthy monkeys. n=19 and 21 for EPC and EMP, respectively.

| **EPC or EMP #/mL** | **r value (FMD)** | **p value (FMD)** | **r value**  **(age)** | **p value (age)** |
| --- | --- | --- | --- | --- |
| EPC (CD45-CD31+CD34+VEGFR2+) | 0.31 | 0.19 | -0.16 | 0.55 |
| EMP(CD45-CD42a-CD31+) | -0.05 | 0.84 | 0.10 | 0.68 |
| EMP(CD45-CD42a-CD144+) | -0.26 | 0.26 | 0.33 | 0.15 |
| EMP(CD45-CD42a-CD144+VEGFR2) | -0.22 | 0.35 | 0.28 | 0.21 |
| EMP(CD45-CD42a-CD62E+) | 0.26 | 0.25 | -0.29 | 0.20 |
| EMP(CD45-CD42a-VEFGR2+) | -0.11 | 0.63 | -0.01 | 0.98 |
| EMP(CD45-CD42a-VEGFR2+CD62E+) | 0.28 | 0.22 | -0.23 | 0.32 |
| EMP(CD45-CD42a-CD106+) | -0.35 | 0.12 | 0.07 | 0.76 |
| EMP(CD45-CD42a-Annexin V) | -0.02 | 0.94 | -0.01 | 0.97 |
| EMP(CD45-CD42a-CD54+) | 0.23 | 0.31 | -0.13 | 0.57 |
| EMP(CD45-CD42a-CD105+) | 0.37 | 0.10 | -0.33 | 0.14 |
