# Supplementary figures and images for: Effects of age on noninvasive assessments of vascular function in nonhuman primates: implications for translational drug discovery
Source: J Transl Med. 2013 Apr 22;11:101. doi: 10.1186/1479-5876-11-101 (PMC3644259; doi:10.1186/1479-5876-11-101)

Figure S1

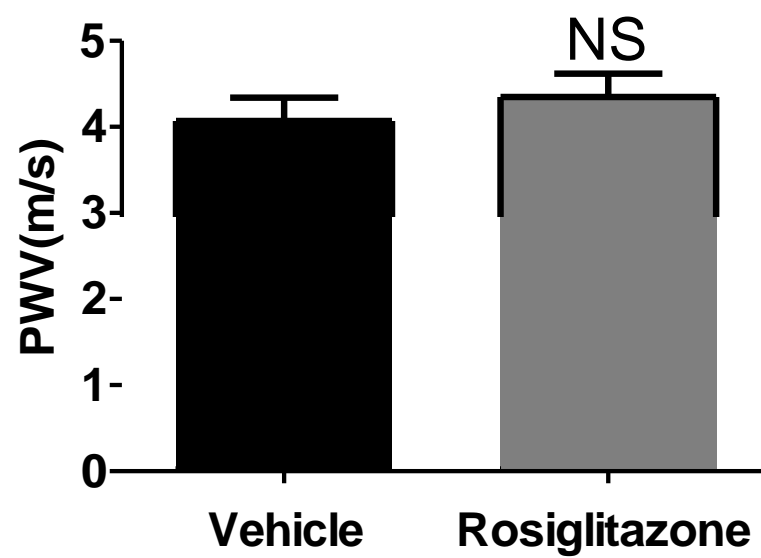

Figure S2

A

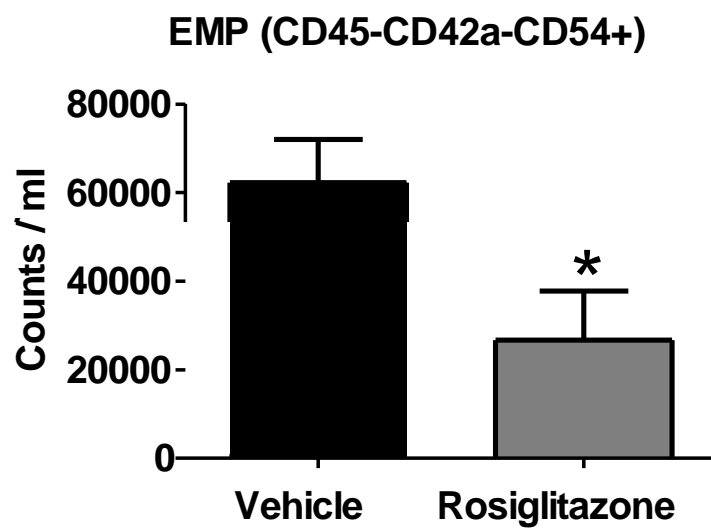

B

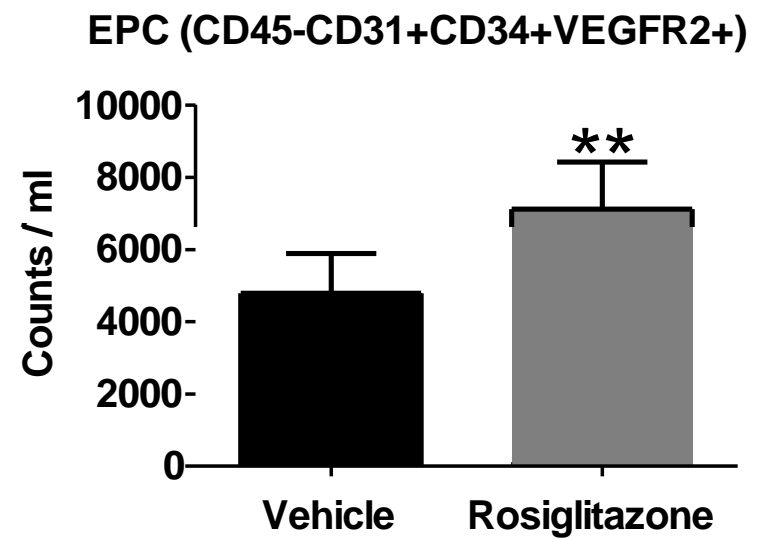

Supplement: Additional file 3: Figure S1 — Rosiglitazone does not affect PWV in NHPs. n = 6, p = 0.22 (age adjusted). NS: non-significant. Figure S2. Rosiglitazone results in favorable changes in circulating numbers of EMPs (CD45-CD42a-CD54+) (A) and EPCs (CD45-CD31 + CD34 + VEGFR2+) (B) in euglycemic, senescent monkeys. n = 5, *: p < 0.05; **: p < 0.01. [file 1479-5876-11-101-S3.pdf]
