# Supplementary material for: Effects of age on noninvasive assessments of vascular function in nonhuman primates: implications for translational drug discovery
Source: J Transl Med. 2013 Apr 22;11:101. doi: 10.1186/1479-5876-11-101 (PMC3644259; doi:10.1186/1479-5876-11-101)
Supplement: Additional file 4: Table S3 — Effect of rosiglitazone treatment on blood biochemistry and LPS-stimulated TNFα release. No significant difference between the two groups, n = 5 (vehicle) or 6 (rosiglitazone). Kglc, 5-20: slope of the disappearance of glucose 5-20 min after dosing; AUC0-30: area under the curve for glucose or insulin (0-30 min). [file 1479-5876-11-101-S4.doc]

**Table S3**: Effect of rosiglitazone treatment on blood biochemistry and LPS-stimulated TNF-α release. No significant difference between the two groups, n=5 (vehicle) or 6 (rosiglitazone).

|  | **Vehicle** | **Rosiglitazone** |
| --- | --- | --- |
| **Fasted Baseline Values:** |  |  |
| Glucose (mg/dL) | 63 ± 2.3 | 62 ±3.0 |
| Insulin (uU/mL) | 7 ± 0.7 | 11 ± 2.6 |
| Total cholesterol (mg/dL) | 85±8.5 | 85±8.2 |
| HDL (mg/dL) | 44±5.5 | 40±5.0 |
| LDL (mg/dL) | 34±4.3 | 33±4.9 |
| Triglyceride (mg/dL) | 110±26 | 115±21 |
| **IVGTT Results:** |  |  |
| Glucose response (AUC_0-30_) | 1962 ± 157 | 1947±113 |
| Insulin response (AUC_0-30_) | 1186 ± 152 | 1484±185 |
| K_glc_ value | 3.6±0.3 | 3.6±0.3 |
| **LPS stimulated TNFα**(pg/ml): | 5139±716 | 5143±741 |

K_glc, 5-20_: slope of the disappearance of glucose 5-20 minutes after dosing; AUC_0-30_: area under the curve for glucose or insulin (0-30 minutes).
